# A Qualitative Exploration of Patient and Public Involvement Experiences in Core Outcome Set Development: Insights From Public Partners and Research Stakeholders

**DOI:** 10.1111/hex.70882

**Published:** 2026-11-04

**Authors:** Shaelynn Hsu, Caoimhin Mac Giolla Phadraig, Tracey Scott, Mandy Daly, Carolyn Akintola, Andrew Geddis‐Regan

**Affiliations:** ^1^ Department of Child and Public Dental Health Dublin Dental University Hospital, School of Dental Sciences, Trinity College Dublin Dublin Ireland; ^2^ Irish Neonatal Health Alliance Bray Ireland; ^3^ Former Family Carer Dublin Ireland; ^4^ Manchester University NHS Foundation Trust, Honorary Senior Lecturer in Dentistry University of Manchester Manchester England

**Keywords:** core outcome set, core outcome set methods, patient involvement, patient participation, public participation, qualitative research, trial methodology

## Abstract

**Introduction:**

Patient and public involvement (PPI) in research engages patients and the public in all aspects of the research process, ensuring that the perspectives of those directly affected by healthcare systems are integral to decision‐making processes. PPI is recognised as central to the development of Core Outcome Sets (COS), standardised collections of outcomes that should be reported in all clinical trials. Despite agreement on the importance of PPI, limited evidence exists on the experience of PPI across partners and research stakeholders. This study explored experiences of PPI during the development of a Core Outcome Set (COS) for dental behaviour support (DBS), with recommendations for better practice.

**Methods:**

Qualitative data were collected through semi‐structured group discussions and individual interviews with PPI partners and key research stakeholders who designed, coordinated, or worked alongside the PPI process. Transcripts were analysed using iterative reflexive thematic analysis. Themes were developed and refined through continuous engagement with PPI partners, who provided feedback to shape interpretation and ensure authenticity. Data management and coding were conducted using NVivo (QSR International Pty Ltd, Version 12).

**Results:**

Four themes captured partners' experiences of PPI within COS development. These highlighted the importance of managing expectations, creating a supportive and inclusive environment, and fostering opportunities for shared learning and reflection. Ten participants were interviewed, including PPI partners and key research stakeholders. Partners described how meaningful involvement changed their understanding of research and strengthened the partnership between researchers and the public. Practical recommendations were shared for future PPI engagement in COS studies.

**Conclusion:**

Effective PPI in COS development requires clear role definition, inclusive collaboration, and ongoing reflexivity. A dedicated PPI Coordinator plays a vital role in supporting communication, trust, and PPI partner empowerment, ensuring participants are valued as co‐developers rather than passive reviewers.

**Patient and Public Contribution:**

PPI partners initiated this study, participated as interviewees, supported data interpretation, and co‐authored this paper.

## 1 Introduction

Patient and Public Involvement (PPI) refers to research that is carried out “with” or “by” members of the public, rather than “to”, “about”, or “for” them” [1]. In practice, PPI involves patients, carers, and members of the public working in partnership with researchers across all stages of the research process, including priority setting, study design, data collection, analysis, and dissemination [2]. By embedding these perspectives, PPI aims to improve the relevance, quality, and impact of research, inform healthcare outcomes, promote equity, and ensure that services are responsive to the needs and priorities of the communities they serve.

Core Outcome Sets (COS) are an agreed set of outcomes that clinicians, researchers and the public agree should be reported in all clinical trials within a particular area of clinical practice [3]. This is a very important step in standardising and comparing research, reducing research waste and enhancing patient outcomes across health conditions. The COMET (Core Outcome Measures in Effectiveness Trials) Initiative has laid out standards for COS development that prioritise inclusive, consensus‐driven processes that involve all relevant stakeholders, including patients, carers, and the public [4]. This involvement should take two forms. Firstly through *participation*, whereby patients and the public can take part in the consensus process and contribute critical insights into what constitutes a successful or acceptable outcome, often including emotional, relational, and functional aspects that may not be fully captured by clinicians or researchers alone [5]. Separately, patients and the public can contribute as research partners, through *inclusion*, e.g. as PPI partners. In this role, PPI partners can share roles in design, oversight and dissemination with other members of the research team. This study explores the *experiences* of such PPI partners in a recent COS development study called BeSiDe‐COS, but not the *impact* of PPI on a COS itself. That is reported separately (study under review).

For context, the aim of *BeSiDe‐COS* was to standardise outcomes in the field of Dental Behaviour Support (DBS). DBS includes a range of psychological, environmental and pharmacological interventions designed to help individuals cope with dental procedures and successfully engage in dental care [6]. To give a flavour, interventions include distraction, cognitive behavioural therapy, sedation, and, general anaesthesia [7, 8, 9, 10, 11, 12, 13]. These interventions aim to make dental care more acceptable and accessible to patients who may experience significant anxiety, behavioural challenges, or other barriers to dental treatment. As such, the outcomes are most relevant to people with anxiety, disabilities and at extremes of age. Given the wide variety of intended outcomes and interventions in this field [14, 15], it was essential to develop a COS to evaluate and compare the effectiveness of DBS techniques.

Recognising the challenges of applying COMET standards for inclusion in a field with particular relevance to individuals with dental anxiety, disabilities, and those at the extremes of age, the BeSiDe‐COS team engaged a diverse group of Patient and Public Involvement (PPI) partners. To promote inclusivity and ensure representation of under‐served and marginalised perspectives, partners were actively recruited through disability representative and advocacy organisations. Examples included patient advocacy, dementia and disability organisations. As a reasonable accommodation, flexible options were offered for partner input. A dedicated PPI lead coordinated PPI partner activity from protocoling to final consensus meetings. This collaborative effort ensured that the outcomes selected reflected the perspectives of all stakeholders, including patients, clinicians, and researchers.

This study addresses an important gap in the COS and PPI literature by exploring how PPI partners and other stakeholders experienced involvement in COS development. This study responds to a suggestion from the BeSiDe‐COS PPI group themselves, who recommended that their experiences of involvement in the BeSiDe‐COS project be documented and shared as a valuable output. In keeping with the principles of PPI, the Study Management Group (SMG) agreed to co‐create a study reflecting their experiences and recommendations. It is hoped that these insights into the relational, ethical, and emotional dimensions of patient and public involvement will be informative to others designing and engaging in PPI in COS research and more broadly. Research into its effectiveness and impact remains limited [2]. This may in part be due to a lack of emphasis on reporting the impact of PPI involvement in COS reporting standards [16]. While there is emphasis on how to involve patients and the public in COS development [5, 17], less is known about the experiences of these partners. Understanding the experiences of PPI partners is crucial to refining the methods and practices of involving patients and the public in future COS development and broader health research.

## 2 Aim

This study aimed to explore perspectives of PPI involvement in the BeSiDe‐COS study and to generate recommendations for strengthening future PPI in core outcome set (COS) development. While the primary focus was the experience of PPI partners, data were also collected from key research stakeholders who designed, coordinated, or worked alongside the PPI process, to provide a fuller account of how involvement was experienced and enacted. This study was not intended to describe the impact of PPI within the COS study itself, which is reported in the main COS study (*in press*). Instead, it represents a supplementary qualitative study of stakeholder perspectives on that process. The findings and insights are intended primarily for researchers, trial methodologists, COS developers, and PPI leads engaged in applied health research, providing guidance on best practices and strategies for meaningful and impactful PPI integration.

## 3 Materials and Methods

### 3.1 Design and Approach

This study employed a qualitative design employing semi‐structured focus groups and interviews. Reflexive thematic analysis was used, with explicit attention to insider positionality and reflexive engagement, recognising the impact of some members of the research team having participated in the wider study context [18]. The study was conducted within a social constructivist paradigm, acknowledging and embracing the fact that knowledge and meaning are constructed through social interactions and shared experiences [17]. The social interactions informing knowledge generation arose within and between stakeholders in the BeSiDe‐COS PPI group, the SMG, and the SAG. The SMG was responsible for the day‐to‐day management of the study and met regularly, while the SAG comprised approximately ten professionals, including clinicians, researchers, methodological experts, and a PPI representative, who provided strategic oversight, advised on the protocol, supported study delivery, and contributed to dissemination. Members of these groups were directly approached and were the population from which purposive sampling was undertaken.

The study protocol received ethical approval from Trinity College Dublin (Ref No: 4684). Data were collected through focus groups and interviews conducted online and audio‐recorded. Informed written consent was gained before interviews were conducted remotely by video using Zoom (Zoom Video Communications Inc. (2024) v.5.17.10). Data collection was led by the PPI coordinator, a second‐year dental student & SMG member who had qualitative research training during her undergraduate research work and was supported in interviewing by experienced co‐authors with doctoral‐level training in qualitative methods. Data collection was conducted via Zoom‐based focus groups between July 1st and July 9th, 2025. A semi‐structured topic guide, developed in consultation with the PPI group, was used to prompt discussion around PPI roles, experiences, and perceived value. Focus groups lasted 45‐60 min, were audio‐recorded, and transcribed verbatim. The focus group and interview recordings were transcribed verbatim by SH using Microsoft Word. The audio data was immediately deleted from the Trinity‐controlled Zoom cloud once transcription was complete.

A pragmatic approach was adopted for both sampling and data generation, reflecting the diverse roles individuals had played in the development of the Core Outcome Set (COS), which made them eligible for inclusion. Recruitment was facilitated by existing relationships: the PPI coordinator, who conducted the interviews, had an established rapport with the PPI group and maintained a professional connection with the SMG. This existing relationship may have facilitated open discussion but may also have influenced what participants felt comfortable sharing. Notably, the study itself was proposed by some PPI partners, further strengthening engagement. Due to logistical challenges in coordinating all participants for group discussions, individual interviews were conducted when necessary to ensure that each participant's experiences and perspectives were fully captured.

Because the eligible participant pool was small and predefined, the aim was not data saturation but to capture the range of perspectives available across those involved in the BeSiDe‐COS PPI process. Therefore, the goal was not to reach saturation but to capture the diversity of experiences within the available group as comprehensively as possible.

### 3.2 Data Analysis and Reflexivity

An inductive reflexive thematic analysis was used to analyse the qualitative data. This iterative approach centred participants' perspectives while recognising the researchers' active role in meaning‐making. A degree of autoethnographic analysis was also integrated, as members of the research team were themselves involved in the broader study context. This dual role required careful reflexivity to balance insider insight with critical distance [19].

Two researchers (SH and AGR) independently conducted the initial analysis. They began by repeatedly reading the transcripts to familiarise themselves with the data, followed by the independent development of initial codes generated inductively, using NVivo (QSR International Pty Ltd., Version 12). Codes were not guided by predefined categories but were directly identified from the data. These researchers then met to compare and discuss codes and initial thoughts on preliminary categories. A coding tree was intentionally not used. Categories were refined collaboratively into overarching themes that captured patterns of shared meaning.

Summaries of initial categories and associated key quotes from the dataset were shared with the same participants who had generated the data and were invited to comment on these and offer an opinion on their alignment with their experiences. Further refinement arose following this engagement process and iterative discussion with further members of the BeSiDe‐COS Team (CMGP).

Given the research team's embedded position, reflexivity played a central role throughout. The researchers maintained awareness of how their personal experiences, assumptions, and prior involvement could influence interpretation. This was supported by regular analytic discussions, including with the PPI authors (TS, MD, CA), ensuring transparency and critical engagement. Final themes were refined considering the research objectives and the team's dual positionality, offering a nuanced interpretation that reflected both participant accounts and researcher‐participant perspectives.

This study is reported in accordance with the COREQ checklist for qualitative research.

## 4 Results

Ten individuals contributed to focus groups or interviews between July 1st 2025 and July 9th 2025. The sample included: five PPI partners and five stakeholders (See Table 1 for descriptions). Four themes were identified, following our iterative review of the data. These are summarised below.

**Table 1:** Participant roles in the BeSiDe‐COS study and qualitative study.

Informant	Gender	Country	Role in BeSiDe‐COS	Role in qualitative study	Role in analysis and interpretation
P1	Female	Canada	PPI Lead, SMG member	Interview coordinator, protocol development, informant, transcription	Primary
P2	Male	UK	Co‐PI, SMG member	Protocol development, informant	Primary
P3	Male	Ireland	PI, SMG member	Protocol development, informant	Secondary
P4	Female	Ireland	PPI partner	Informant	Secondary
P5	Female	Ireland	PPI partner (patient advocate)	Informant	Secondary
P6	Female	Ireland	PPI partner (parent/carer)	Informant	Secondary
P7	Male	UK	PPI partner	Informant	None
P8	Female	Ireland	SAG member	Informant	None
P9	Female	Malaysia	SAG member	Informant	None
P10	Male	Saudi Arabia	SAG member	Informant	None

*Note:* Primary indicates leading coding and interpretation; *Secondary* indicates contribution to theme refinement and interpretation.

Abbreviations: PPI, patient and public involvement; SAG, study advisory group; SMG, study management group.

### 4.1 Managing Roles and Expectations

Several factors contributed to the perceived success of PPI in this COS development project including the appointment of a dedicated PPI lead role, and clarity of panellist expectations through collaboratively developed Terms of Reference. The PPI panel also set ambitious expectations for meaningful involvement. Though initially challenging for the SMG, ultimately this expectation elevated the quality and authenticity of the PPI process.

To begin, the SMG appointed a specific PPI lead, whose role was to build and coordinate the PPI panel. The lead was energetic and committed. This was deemed critical:

if it wasn't for the PPI coordinator's energy, I think we would have really struggled, really struggled.

‐P3, PI

The PPI lead was proactive in engaging PPI panellists. A communication strategy was outlined with the PPI panel at the first meeting, outlining what to expect by way of role and medium. PPI panellist preferences were explored. The lead ensured she was accessible to panellists throughout the process. This assured clear expectations and clarity of roles, which allowed panellists to feel safe:

… So you have a point of contact. You know, somebody that anybody from the PPI panel can go to, and I think that is a key component of success, … you're bringing in public to an arena where they are feeling vulnerable, where they may not understand why they're here.

‐P5, patient advocate

From the outset, the SMG made conscious efforts to clarify what was expected from PPI partners. The terms of reference (ToR) – See Supplemental Materials ‐ were the first item on the agenda and these were explicit in the exact roles of the group. PPI panellists co‐created their ToR, meaning that they decided their own roles with clarity:

Everything was beautifully explained as well, both in writing and in person. That makes a big, big difference. I felt comfortable what you're doing and you didn't feel: Oh, my God, am I in too deep here? Or what the heck am I doing here?

‐P4, PPI partner

From the outset, the PPI partners shared their expectations with SMG. For example, they wished to co‐create resources such as protocol, surveys and recruitment tools – rather than simply comment on. They also wanted a role in recruitment of patient and carer participants. These expectations were surprising and challenging for the SMG but the fact that PPI partners set a high standard ensured that the SMG delivered:

So if my expectations were high, my expectations were met, because I certainly was very familiar with the role coming into this, which is something I often struggle with in in my job. People need PPI. They want PPI, but sometimes they don't know what to do with PPI ‐ what PPI is meant to look like…

‐P5, patient advocate

### 4.2 Building an Inclusive and Supportive Environment

An inclusive environment was purposely established through intentional and resource‐intensive efforts. As part of this inclusive approach, the SMG prioritised diversity and inclusivity in forming the PPI panel. In particular, people with disabilities and carers were sought, given the specific salience of DBS for these groups. Additionally, the team wanted to avoid sampling from convenient pools of patients with whom they have good and close relationships, fearing that the PPI process would be skewed:

…the qualitative research that generates core outcome sets tends to [include] the dentist's or doctor's favourite patient… You end up with people who've had good outcomes, who haven't put in complaints, you know ‐ you end up with, like, the happy people.

‐P3, PI

While efforts were made to achieve a diverse group of PPI partners, who they did not know and who wanted to collaborate from abroad, this was also challenging:

But it was really important to have that mix‐ the mix of different roles, the mix of different nationalities. So, I see a Brazilian, a Dub[liner] and a Canadian. And there was a Brazilian, a Dub[liner] and a Canadian on the panel. So, it goes to show that what we aimed for this cold calling and PPI Panel generation, my God that was difficult.

‐P3, PI

While this was a challenge, the PPI panellists felt that diversity was both achieved and advantageous. The PPI panel considered this diversity a strength:

…in this instance it was quite clear that inclusion and equity were very much highly up the priority list in terms of … the public and patient community.

‐P5, patient advocate

I think, having somebody, or having a number of people at a table with different perspectives, with different levels of experience is really important

‐P5, patient advocate

Regarding communication, the SMG aimed for two‐way communication that fostered understanding among the PPI group and allowed them to feel welcome to communicate their ideas with confidence:

…there was the understanding that the language that was used was very lay

terminology. At no point was anybody's opinion dismissed. Everything was heard.

‐P5, patient advocate

The SMG made efforts to ensure the PPI group could see their impact at every stage. After contributions, they were updated of the changes their input had made to the protocol, recruitment strategy, survey, etc. This meant that panellists were clear that their input was valued, encouraging further input. This open bidirectional communication fostered a sense of safety and trust:

there was no point at any time where my personal experience was, I won't say, dismissed…It was certainly heard, and I think I liked that.

‐P5, patient advocate

There was a definite warmth there. Even when there were big groups, it was as if, look, we're all here for the one purpose.

‐P4, PPI partner

This created a group that felt safe together in alliance with the SMG. The use of subtitled videos to introduce aspects of the project impressed a sense of effort by the SMG to value the PPI panel:

the time that you guys spent in putting together even a video to explain what this is all about, to try to, you know, break that language barrier so people can actually pause. You have subtitles in, you actually, you were very careful.

‐P9, SAG member

Demonstrating trustworthiness and appreciation was important given the potential trauma and vulnerability inherent in engaging in PPI, even in areas as seemingly banal as dental care:

I liked the active listening. I like the very active participation. I like the time that was given to PPIs to talk, and that encouragement, because we all bring a different hat to this table. But the understanding and the recognition that there are different life stories coming to the table and understanding that for some people it's a very traumatic experience.

‐P5, patient advocate

### 4.3 Learning through Doing

Reflecting the complexity and iterative nature of the associated COS development process,

the PPI process was also one which evolved and developed over time, with elements of trial and error. PPI partners were a mix of first timers and experienced contributors. Over the course of the project, an initial sense of trepidation towards the unknown was gradually replaced with confidence:

…I was anxious about it because it's important to do it well, but it's so difficult to do it well. You know, it's so difficult.

‐P2, Co‐PI

This shift was in part achieved by reaching out to professional PPI organisations (eg. PPI Ignite Network) for a gentle steer:

…at the start we were out of our depth and we didn't know what we were doing. So we reached out to experts… to guide us, and I have to say, after our second meeting with… the experts… it was clear that we were well ahead of the curve.

– P3, PI

Participants reflected on both successes and challenge. Over time, steps taken within PPI were refined and amplified, contributing directly to the correct steps for the wider project:

I think now that I've gone through the whole process, like, I think, yeah, just setting up maybe the terms of reference a little bit differently, because I think I also wanted them to be much more involved, maybe, than they even expected in the very beginning. So yeah, maybe setting those expectations from the very beginning. But I think that would have been hard to do for this study, just because we didn't know what to fully expect.

‐P1, PPI lead

### 4.4 Transforming Understandings of Ppi

Views of PPI evolved throughout the project. All involved were somewhat daunted by the task ahead when initiating the COS process. Members of the SMG felt somewhat forced into engaging with PPI to ensure compliance with COMET standards. While at first this was seen as a barrier, this perspective changed over time:

…if I just look back [at] developing the panel, [I felt] oh this is because, COMET are making me do it. I actually think that, especially with this reflection here, you can see all the benefits that we that we reaped and in the core outcome set.

‐P3, PI

Panellists also felt reticent in engaging initially. They also felt compelled to engage:

Initially, I'm saying, What the hell am I doing within this? Right? And then I sat back and I said: Well, you know, I can't complain about services or nothing changing if I don't change it, you know, if you don't see change, you have to be change.

‐P4, PPI partner

During the COS development PPI partners took on very visible roles. They co‐produced and “starred” in information videos to facilitate survey completion. They also co‐presented the Consensus meetings too. The PPI panellists sensed the impact that their status and presence had on the Delphi and Consensus panellists too:

You could see facial expressions changing. Particularly those, maybe from cultures that are not really all that comfortable with disabled people. As you could see that they were thinking,.: “Uh oh, is this what I'm facing, you know. And you could see that definitely there was very much a change in facial expressions.

‐P4, PPI partner

Although initially daunting, involvement in PPI was ultimately described as rewarding. Specifically, a sense of empowerment was described, by partners being given an opportunity to influence care for many from an upstream point:

I loved it. I think it's the largest project I've been involved with that has actually asked for patient opinion, and seem to value that patient or family member or carer participation, particularly when you've got a disability you're so accustomed to services being delivered in the way that the service provider, the institution, the state, wants to provide it, without asking your opinion of how you want services provided:‘ what you want, what you don't want, because choice is very limited in healthcare and anyway when you're disabled, so the choice that you do have needs to at least partly empower you, right? I've been very, very, delighted to have been invited. It was very, very much, a very interesting project.

‐P4, PPI partner

Looking forward, the PPI panellists reflected on the share learning between researchers and PPI panellists. Some clearly stated a sense of pride in their perceived impact on the research team, having taught PPI skills to the researchers and having motivated the research team to raise the standard of PPI involvement both within this COS and into the future:

I like to think that you've taken something away from this, and you know from working with us, and that it will encourage you to do so again in future, and probably encourage some of your colleagues and peers to do so as well, because we know, you know, for those larger projects which are funded. PPI is now almost a blanket, but there are so many out there who don't have the experience of working with us, and honestly, I like to feel I was part of the entire process, and that you know my contribution helped right along the entire way.

‐P5, patient advocate

## 5 Discussion

This study explored experiences of PPI during the development of a Core Outcome Set (COS) for dental behaviour support (DBS), with recommendations for better practice. Given the recognised importance of PPI in COS development [4, 5, 20] and applied health research in general [1, 21], proactively examining partners' experiences provided an opportunity to identify factors that support meaningful involvement and shape future practice. Although engaging PPI partners required additional commitment, their input generated valuable insights to strengthen both the effectiveness and acceptability of PPI in similar research contexts.

A central finding was the importance of clear and transparent communication from the outset. Even when specific activities could not be precisely defined at recruitment, articulating the overall purpose of PPI helped identify partners with relevant lived experience and perspectives. This clarity also enabled individuals to make informed decisions about participation, ensuring commitment once engaged. Recruitment combined personal networks with targeted outreach to PPI and disability organisations (eg. PPI Ignite Network, Irish Platform for Patient Organisations, Science & Industry, and Holland Bloorview Kids Rehabilitation Hospital). Although this approach carried some inherent risk – namely that participating PPI partners might be overly constituted by friends and associates of the research team ‐ clearly defined expectations facilitated smooth onboarding and strengthened a shared understanding between the research team and contributors. Previous studies describe PPI as a complex social activity involving multiple potential roles [22, 23], and our findings support this: early discussion of expectations was crucial for sustaining engagement throughout the study.

Despite the absence of dedicated funding—which is detailed as necessary for optimal PPI [21, 24] —appointing a single point of contact through a PPI lead proved highly effective. This role streamlined communication, reinforced understanding of project aims and supported consistent engagement. Interestingly, the lack of financial support was not detailed as a major barrier by PPI partners or other stakeholders in the COS development process. Instead, a collaborative team dynamic, supported by trust and open communication, was central to meaningful involvement. While funding can facilitate engagement, this finding highlights that the quality of the relationships and communication structures may be equally influential.

Creating an inclusive and supportive environment was also fundamental to ensuring meaningful participation. Active efforts to adapt language, simplify technical content and offer ongoing support helped lower barriers to engagement, regardless of partners' prior research experience. Partners were not passive participants but engaged as co‐thinkers, particularly during planning and Delphi stages. Their input was embedded within the decision‐making process rather than treated as supplementary. This was achieved through feedback bidirectionally supported by the PPI lead, transparent communication and deliberate strategies to build trust. Although some power imbalance is inevitable in research structures, this did not appear to diminish partners' sense of value or impact. Importantly, the idea for this reflective element of the study originated with the PPI panel themselves. Their interest in documenting their experiences demonstrates genuine collaboration and shared ownership, further strengthening the authenticity of their involvement.

While partner experiences were largely positive, the study also highlighted areas for development. The COS was designed to have international relevance, but most PPI partners were English‐speaking participants from high‐income countries. This reflects the study team members' locations but also points to the need for more diverse engagement in future PPI for both COS development and DBS research. DBS interventions are relevant to individuals with varied and often complex needs, including those experiencing health inequalities. As previous work has shown, PPI approaches can be adapted to include individuals with communication or cognitive impairments [25, 26, 27] through flexible methods that ensure their perspectives inform outcomes, even when direct participation in Delphi processes is not possible. Future work should prioritise investment in broader recruitment strategies, flexible participation pathways and dedicated PPI engagement leads to enhance inclusivity and representation.

A further key finding was the significant degree of reciprocal learning across all stakeholder groups. Young. [28], described this active process as co‐learning: a process which arose throughout the COS development process. For some participants, early involvement was accompanied by uncertainty and a sense of being “out of our depth”, reflecting both willingness to engage as well as uncertainty about operationalising PPI. Similar anxieties have been described in other studies [29, 30, 31], but here they became part of a productive and adaptive learning process rather than a barrier. The research team learned both from PPI partners' direct input and from the practical process of integrating their perspectives into the study design. PPI was pivotal in shaping Delphi rounds and necessitated two consensus meetings to finalise the COS, highlighting its real and substantial influence on study outcomes. The process also revealed how outcomes would have differed without PPI, further demonstrating its value. While partners were less explicit in articulating co‐learning, their subsequent reflections confirmed that this was a shared and mutually beneficial process.

The impact of this co‐learning extended beyond the immediate project, influencing how stakeholders viewed PPI in research more broadly. The progression of time, and the associated increase in studies utilising PPI has led to an increasing number of reports on its use, impact, and learning that arises from its enactment [25, 30, 31, 32, 33]. There are fewer case studies related to the dental context, though challenges aligned with those discussed in this project have been summarised in explorations of PPI in various dental discplines [34, 35]. Although some initially regarded PPI as a procedural requirement aligned with COMET guidance [4], by the end of the project it was recognised as a meaningful contributor to both the quality and relevance of the COS. Experiencing its tangible impact first‐hand fostered optimism and willingness to embed PPI in future research, even among those with limited prior experience.

Having proactively explored the views of PPI partners and stakeholders involved in PPI, this study highlights that effective PPI in COS development depends on clear role definition, inclusive communication, and opportunities for shared learning. Even in the absence of funding, intentional planning, trust‐building and genuine collaboration supported meaningful engagement. PPI partners were perceived to shape both the process and the outcomes of the COS, while the co‐learning that emerged transformed how PPI was perceived and valued. These findings point to the importance of moving beyond procedural involvement towards authentic partnership, and of investing in strategies to broaden representation and strengthen the impact of PPI in future research.

### 5.1 Strengths and Limitations

A key strength of this work lies in its origins, as a piece of work proposed by PPI partners. In both the BeSiDe‐COS project and in this reflection on the role of PPI partners in the project, patients and carers were engaged as active contributors rather than passive participants, helping to shape the study design, interpretation of data, and recommendations made.

To account for their potential bias as project team members in various roles, the authors adopted a reflexive practice, regularly reflecting on their positionality, assumptions, and influence on decision‐making regarding the data presented and conclusions drawn herein. This iterative reflexivity aimed to mitigate potential biases within the research process.

However, several limitations should be acknowledged. Not all PPI partners chose to participate in interviews or focus groups, which may have limited the breadth and diversity of perspectives represented in the qualitative data. Additionally, the qualitative data analysis was conducted primarily by members of the research team, some of whom were also interview participants. This dual role may have introduced interpretive bias, despite the team's efforts to remain reflexive and self‐critical throughout. While reflexive discussions provided a mechanism for recognising and addressing these challenges, future work could strengthen credibility by involving PPI partners more directly in data analysis and interpretation, ensuring a fuller integration of patient perspectives.

## 6 Recommendations

Reflecting on the work presented herein, some key recommendations can be made for those seeking to use PPI to support similar applied health research processes:

**Dedicated PPI Coordinator:** A clearly designated PPI lead can support continuity, communication, and trust‐building across the research process.

**Recruit for Balance**: Step beyond familiar networks to build a PPI group with diverse backgrounds and experiences. This process is onerous and uncertain but of value. Recruiting for balance broadens perspectives and strengthens the relevance, inclusivity, and robustness of the final outcome.

**Empower the PPI Group:** PPI partners should be supported to shape how they contribute, communicate, and influence decisions throughout the project. This includes providing supportive resources and opportunities for networking so that partners with lived experience can engage meaningfully and equitably. Empowerment also involves embedding flexibility into the process, allowing stages of co‐production to evolve according to partner needs and preferences.

**Build an Alliance:** Meaningful PPI depends on reciprocal trust, accessible communication, and visible responsiveness to partner input.

## 7 Conclusion

Meaningful patient and public involvement (PPI) requires clear role definition, inclusive relationships, and sustained reflection throughout the research process. The presence of a dedicated PPI Coordinator is critical to facilitating communication, fostering trust, and ensuring that partners are supported and valued as co‐creators rather than passive reviewers. Efforts to respond to participant preferences, provide transparent feedback on how their input influences research decisions, and adapt to evolving relational and contextual dynamics are essential to maintaining authentic and equitable engagement. A further key finding was the significant degree of reciprocal learning observed across all stakeholder groups.

Engagement in the project fostered mutual understanding, with researchers, practitioners, and PPI partners each developing new insights into one another's perspectives, expertise, and constraints. Attitudes towards PPI also evolved over the life course of the project, with many participants reporting a shift from initial uncertainty to a deeper appreciation of its value and influence. These findings highlight that both PPI partners and those facilitating or leading PPI groups should anticipate a dynamic and developmental experience, in which roles, relationships, and expectations mature through sustained interaction and reflection. Looking ahead, future work should prioritise investment in broader and more inclusive recruitment strategies, flexible participation pathways, and dedicated PPI engagement leads to strengthen representation, responsiveness, and the long‐term sustainability of involvement in health research.

## Author Contributions

**Shaelynn Hsu:** conceptualisation, investigation, writing – original draft, methodology, writing – review and editing, software, formal analysis, data curation, validation, project administration. **Caoimhin Mac Giolla Phadraig:** conceptualisation, investigation, methodology, validation, writing – review and editing, project administration, supervision. **Tracey Scott:** conceptualisation, validation, formal analysis, writing – review and editing. **Mandy Daly:** conceptualisation, validation, formal analysis, writing – review and editing. **Carolyn Akintola:** conceptualisation, validation, writing – review and editing, formal analysis. **Andrew Geddis‐Regan:** conceptualisation, investigation, writing – original draft, methodology, validation, writing – review and editing, software, formal analysis, project administration, data curation, supervision, resources.

## Funding

The authors have nothing to report.

## Ethics Statement

School of Dental Science, Research Ethics Committee, Trinity College Dublin Ref No: 4684.

## Conflicts of Interest

The authors declare no conflicts of interest. Some authors held dual roles within the wider study context; however, reflexive practices were employed throughout to ensure transparency and minimise potential bias.

## Supporting information


Supporting File

## Data Availability

Data are available upon request from the authors.
