# Supplementary material for: A Qualitative Exploration of Patient and Public Involvement Experiences in Core Outcome Set Development: Insights From Public Partners and Research Stakeholders
Source: Health Expect. 2026 Nov 4;29(6):e70882. doi: 10.1111/hex.70882 (PMC13732645; doi:10.1111/hex.70882)
Supplement: Supplementary file 1 [file HEX-29-e70882-s001.docx]

Terms of Reference

# Patient and Public Involvement (PPI)

## Purpose:

The patient and public involvement (PPI) contributors are integral to the BeSiDe Core Outcome Set (COS) study, ensuring that the patient and public perspective is included in the design, conduct, and outcomes of the study.

## Key Responsibilities

- Co-design the study, ensuring it aligns with patient and public priorities.
- Co-create study materials, including surveys, outcome lists, and research processes.
- Participate in discussions to ensure that the outcomes identified are meaningful to patients and the public.
- Co-plan consensus meetings to validate and refine the outcomes from the patient perspective.
- Act as ambassadors for the study, including dissemination of publication regarding PPI process and outcomes.

## Membership

- Composed of patients, family members, and individuals with personal or familial experience of dental behaviour support techniques.
- Members will represent diverse perspectives, including those who have experience with general dentistry, general anaesthesia, sedation, CBT (Cognitive Behavioral Therapy), and other behaviour support techniques.
- Meetings will be held at key points in the study, particularly during the co-creation stages.

## Involvement Approach

- Members will be actively engaged throughout the study, following the Public and Patient Involvement (PPI) guidelines from the COMET initiative.
- It is expected that four online meetings will be held over the 18 month tenure of the PRP panel.
- Alternative options to engage will be made available to include individuals who may otherwise be excluded in research, such as those with communication impairments or other difficulties that might prevent traditional participation.
- Participation will be supported with accessible meeting formats and clear communication to ensure everyone can contribute fully.

## Reporting

PRPs will provide their insights and feedback to the Study Management Group (SMG) and Study Advisory Group (SAG), ensuring that public and patient input is considered at all stages of the study. A member of the PPI panel may sit on the SAG as agreed.

Delphi Survey Instructional Video:

<https://drive.google.com/file/d/1MLg0Ba09-CqtZo2p7VZaYT5eNbDF4Orr/view?usp=sharing>
